# Correlates of foot pain severity in adults with hallux valgus: a cross-sectional study

**DOI:** 10.1186/1757-1146-7-32

**Published:** 2014-06-28

**Authors:** Sheree E Hurn, Bill T Vicenzino, Michelle D Smith

**Affiliations:** 1 Queensland University of Technology, School of Clinical Sciences, Kelvin Grove, QLD 4059, Australia; 2 Queensland University of Technology, Institute of Health and Biomedical Innovation, Kelvin Grove, QLD 4059, Australia; 3 The University of Queensland, School of Health and Rehabilitation Sciences, Division of Physiotherapy, St Lucia, QLD 4072, Australia

## Abstract

**Background:**

Hallux valgus (HV) is highly prevalent and associated with progressive first metatarsophalangeal joint subluxation and osteoarthritis. The link between structural HV deformity and foot pain is unclear. This study investigated possible explanatory factors surrounding foot pain in HV, including radiographic HV angle and signs of joint degeneration.

**Methods:**

Participants were 60 adults (53 female) with HV aged 20 to 75 years. Participant demographics and a range of radiographic, clinical and functional measures were considered potential correlates of foot pain. Self-reported foot pain (visual analogue scales and a dichotomous definition) was considered the dependent variable. Multivariate modelling was used to determine which characteristics and measures explained pain, with univariate analyses first used to screen potential variables.

**Results:**

Approximately 20 to 30% of the variance in foot pain associated with HV could be explained by patient characteristics such as poorer general health status, lower educational attainment and increased occupational physical activity levels, in combination with some dynamic physical characteristics such as hallux plantarflexion weakness and reduced force-time integral under the second metatarsal during gait. Neither increasing lateral deviation of the hallux (HV angle) nor presence of first metatarsophalangeal joint osteoarthritis was associated with foot pain.

**Conclusions:**

This study shows that passive structural factors, including HV angle, do not appear to be significant correlates of foot pain intensity in HV. Our data demonstrate the importance of considering patient characteristics such as general health and physical activity levels when assessing foot pain associated with HV.

## Background

Hallux valgus (HV) is a highly prevalent forefoot deformity [1], presenting with lateral deviation of the first toe and progressive subluxation of the first metatarsophalangeal (MTP) joint. HV is often associated with first MTP joint osteoarthritis (OA) [2] and has been linked to impaired physical function [3,4] and poorer general health and quality of life [3-5]. However, there have been contradictory reports regarding the link between foot pain and HV [4,6-10].

Pain associated with HV is often attributed to local mechanical stimuli [11] or degenerative processes within the first MTP joint-sesamoid complex [2]. It could therefore be hypothesised that more severe deformity may be associated with more severe pain, and one previous study has found a positive association between self-reported HV severity and increasing pain levels [4], although being a population survey radiographic measures were not used. Interestingly, radiographic studies of other lower limb conditions have found no association between pain levels and radiographic measures of knee OA [12] or first MTP joint OA [13]. No previous study has comprehensively investigated the range of foot and ankle characteristics that might explain foot pain symptoms in HV, including plantar pressures [14,15], plantar hyperkeratosis [16], muscle weakness [3] and footwear [17].

In addition to foot and ankle factors, pain severity may be influenced by demographic, cognitive and lifestyle factors, with previous studies showing foot pain to be worse in females, older people, those with higher body mass index (BMI) and lower educational attainment [3,7,13,18-20]. A study of pain symptoms in HV should therefore investigate local factors as well as patient characteristics.

This cross-sectional study examined potential explanatory factors surrounding foot pain in HV, including radiographic HV angle and signs of OA, as well as foot and ankle factors and participant characteristics. Differences between participants with and without disabling foot pain were also investigated.

## Methods

### Participants

Participants with HV (defined as a radiographic HV angle greater than 15 degrees) were recruited through community advertisements. Potentially eligible volunteers aged 20 years and older were screened for the following exclusion criteria: history of foot or ankle surgery or fractures, inflammatory disease, neurological conditions and history of falls. Because radiographs were required for this study, pregnant or breastfeeding women were also excluded. In order to exclude cases with concomitant hallux limitus, HV participants were required to have a minimum of 50 degrees passive dorsiflexion at the first MTP joint [21]. Ethical approval was gained from The University of Queensland Medical Research Ethics Committee, and all participants gave written informed consent prior to involvement in this study.

### Measurement procedure

#### Foot pain

Foot pain intensity was measured on a 100 mm visual analogue scale (VAS), with 0 mm described as “no pain” and 100 mm described as “worst pain ever.” Participants were asked to indicate their worst level of foot pain and average level of foot pain experienced over the past four weeks. The examiner marked the pain location(s) on a diagram. To further describe foot pain as it relates to functional disability, participants completed the Manchester Foot Pain and Disability Index [22]. If one of the 10 functional limitation items was reported to occur on “most/every day”, participants were considered to have “disabling foot pain” [23].

#### Participant characteristics

Demographic data including sex, age, ethnicity, and level of education were obtained via questionnaire. Height and weight were recorded and BMI was calculated. General health was assessed using the SF-36v2® Health Survey general health subscale (score range 0 – 100, with higher scores indicating better general health) [24]. Habitual physical activity levels were assessed using the Baecke questionnaire to calculate a work, sport, and leisure index [25].

#### Assessment of HV

Bilateral weight bearing dorsoplantar radiographs were obtained for all participants using a standardised procedure (tube to film distance 100 cm, angled 15 degrees from vertical). Alignment between the first metatarsal and proximal phalanx, or HV angle, was measured using computer software developed for telemedical applications [26]. Dorsoplantar radiographs were then examined for presence of OA at the first MTP joint [27,28]. Severity of HV deformity was also graded clinically using the Manchester Scale [29,30] and both feet were examined for the presence of forefoot calluses, recorded by the examiner as “present” or “absent.”

#### Pressure-pain threshold

Pressure-pain threshold was measured at the medial and plantar aspects of the first MTP joint to obtain a quantifiable measurement of mechanical hyperalgesia. A digital pressure algometer (Somedic AB, Farsta, Sweden) was used to measure pressure, which was applied at a rate of 40 kPa/s via a rubber-tipped probe (area 1 cm^2^). Participants were asked to indicate when the sensation of pressure changed to onset of pain. An average of three measurements from each site was used for analysis.

#### Foot posture and joint mobility

Foot posture was assessed using the six-item Foot Posture Index [31-33], which provides a score ranging from -12 to +12, indicating a supinated (negative) or pronated (positive) foot type. Dorsal arch height (DAH) and midfoot width (MFW) were measured in weight bearing and non-weight bearing using a protocol previously described [34]. The difference between weight bearing and non-weight bearing DAH and MFW provides an indication of foot mobility, and foot mobility magnitude (FMM) is a composite measure representing change in both DAH and MFW. Generalised joint hypermobility was assessed using the nine-point Beighton scale [35]. The weight bearing lunge test was used to assess dorsiflexion range of motion (ROM) at the ankle joint [36] and passive dorsiflexion ROM at the first MTP joint was assessed in non-weight bearing using a small goniometer [21].

#### Hallux plantarflexion and abduction strength

Hallux plantarflexion and abduction strength were evaluated using 50 kg load cells (GK 2126-50, Gedge Systems, Melbourne, Australia) mounted in a custom-built frame [37]. Participants performed three maximum isometric voluntary contractions in each direction while seated, and the maximum force achieved over the three trials was used for analysis. For hallux abduction, the load cell sensor was positioned so that it was just touching the medial aspect of the hallux in each participant’s resting position. Participants were then instructed to “spread their toes apart sideways.” All participants were able to produce some movement of the hallux given this instruction, although the amount of control to abduct the hallux towards the sensor varied greatly between participants, especially those with moderate to severe HV deformity. The paper grip test was used as a clinical measure of hallux plantarflexion strength [38,39]. Participants completed three trials of three seconds, and a pass was recorded if the individual could hold the paper under the hallux against resistance for all trials.

#### Plantar pressures

The Pedar-X® system (Novel_gmbh_, Munich, Germany) was used to evaluate in-shoe plantar pressures. Insoles were fitted in sports shoes or walking shoes provided by the participants who completed five walking trials at a self-selected comfortable speed along a 10 metre walkway. Analysis was based on data from an average of 23 steps over the 5 trials. Five forefoot regions were identified using a relative mask based on prior work by Putti et al. [40]: hallux, lesser toes, first metatarsal, second metatarsal, third to fifth metatarsals. In each region four different parameters were evaluated: peak pressure (kPa), pressure-time integral (kPa*s), maximum force (%BW) and force-time integral (%BW*s).

#### Footwear assessment

In addition to the shoes worn for Pedar® analysis, participants were asked to bring the two pairs of shoes they wore most frequently. Shoes worn to the examination session were assessed for relative heel height using methods previously described [41]. Forefoot ball width was determined using digital callipers to measure the horizontal distance across the widest point of the MTP joints. The callipers were then used to measure the shoe across the widest point of the forefoot, and relative ball width was calculated as the difference between these two measures. Finally, participants were asked: “Have you ever worn shoes with heels two inches high or greater?” (Yes/No) and “How often do you currently wear this style of shoe?” (Never, Seldom, Sometimes, Often, Always) [41].

All measurements were obtained by the principal examiner. Intrarater reliability for several of these measurement methods has been discussed previously [37]: very good reliability (ICCs ≥ 0.90) was found for footwear assessment and radiographic HV angle measurement, good reliability (ICCs ≥ 0.75) was found for pressure-pain threshold at the first MTP joint and hallux plantarflexion strength, while hallux abduction strength measures were moderately reliable (ICC = 0.73) [42].

### Sample size determination

Sample size calculations based on preliminary data analysis indicated that a sample size of 54 would have 90% power to detect a significant effect of HV angle when added to a model with five other predictor variables (alpha level = 0.05; R^2^ change = 0.05). With a 10% allowance for missing data, a sample size of 60 was obtained for this study.

### Statistical analysis

For variables measured bilaterally, only the right or left foot was chosen for analysis [43] based on the greater radiographic HV angle (28 right feet and 32 left feet). Data were screened for normality of distribution, and continuous variables showing a skewed distribution were transformed prior to analysis using an appropriate transformation (log, square or inverse). Means, standard deviations (SD) and frequencies were calculated for the entire sample and for those with and without disabling foot pain [23]. Differences between participants with and without disabling foot pain were investigated using independent t-tests for continuous variables and Chi-squared tests for categorical variables. The level of significance for these tests was *p <* 0.05.

A two-step approach was used to examine multivariate correlates of foot pain intensity (worst and average foot pain VAS). First, univariate analyses were performed to investigate potential associations between average or worst foot pain VAS and each independent variable. Pearson’s or Spearman’s correlations were used for continuous variables, and independent t-tests were used for dichotomous variables. Variables that were categorical were analysed using a one-way analysis of variance with the categorical variable as the grouping variable. Potential predictor variables included the following continuous variables: age, BMI, activity level (work, sport and leisure indices), general health (SF-36v2®), HV angle, pressure-pain threshold, ankle lunge test, first MTP joint dorsiflexion, difference between weight bearing and non-weight bearing DAH and MFW, FMM, plantar pressure parameters (peak pressure, pressure-time integral, maximum force, force-time integral), and footwear measurements (relative heel height, relative ball width). Dichotomous and categorical variables included: sex, ethnicity, education, history and frequency of wearing high heeled shoes, Manchester Scale, presence of forefoot callous, first MTP joint OA, and the paper grip test. The Foot Posture Index and Beighton joint hypermobility score were considered ordinal scales and therefore Spearman’s rho was used to investigate univariate associations with pain VAS, and the Wilcoxon rank-sum test was used to examine differences between those with and without disabling foot pain. Second, variables found to be potentially associated with worst or average foot pain VAS (significance level for screening, *p <* 0.1) were entered into a series of multiple linear regression models on the basis of the strength of their univariate associations. Change in the amount of variance (R^2^), standardised beta weights and *p*-values were examined to determine if variables made a significant independent contribution to the model, and independent variables were retained in the model if *p* ≤ 0.05. Finally, radiographic HV angle was forced into the model to investigate any possible contribution of HV severity to foot pain. Regression models were checked for multicollinearity and normality of residuals. All statistical analyses were conducted using Stata version 10 (StataCorp LP, College Station, TX).

## Results

Participants were 53 women and 7 men with HV, aged 20 to 75 years. HV angles of participants ranged from 15.5 to 54.4 degrees. All participant characteristics (frequencies, means and SDs) are presented in Table 1.

**Table 1:** Participant characteristics and foot and ankle characteristics in those with and without disabling foot pain

	**Total (n = 60)**	**Disabling foot pain (n = 16)**	**No disabling foot pain (n = 44)**	**MD (95% CI) or *p *** **value**
**Participant characteristics**				
Sex (n female)	53	15	38	*p* = 0.43
Age (years)	51.5 ± 15.1	56.6 ± 12.0	49.7 ± 15.7	6.9 (-1.8 to 15.6)
BMI (kg/cm^2^)	25.1 ± 4.3	25.4 ± 3.1	25.0 ± 4.6	0.3 (-2.2 to 2.8)
Ethnicity (n Caucasian)	50	12	38	*p* = 0.55
Education (n)^a^				
High school	13	4	9	*p* = 0.34
Trade/diploma/certificate	15	6	9	
Degree	17	2	15	
Postgraduate	14	4	10	
General health score (SF-36v2)	78.7 ± 14.9	72.3 ± 13.6	81.0 ± 14.8	-8.8 (-17.3 to -0.29)*
Physical activity (score range 1 to 5)				
Work index	2.63 ± 0.35	2.75 ± 0.29	2.58 ± 0.37	0.17 (-0.04 to 0.37)
Sport index	2.68 ± 0.90	2.48 ± 0.88	2.75 ± 0.91	-0.26 (-0.79 to 0.27)
Leisure index	2.86 ± 0.61	2.85 ± 0.75	2.86 ± 0.56	-0.01 (-0.37 to 0.35)
Footwear examination^b^				
Relative heel height > 25 mm (n (%))	8 (13.8%)	2 (13.3%)	6 (14.0%)	*p* = 0.95
Relative ball width (mm)	4.7 ± 4.6	5.0 ± 3.3	4.6 ± 5.0	0.5 (-2.4 to 3.4)
History of wearing high heels (n (%))^a^	29 (49.2%)	8 (53.3%)	21 (47.7%)	*p* = 0.71
Frequency of wearing high heels (n)^a^				
Never	21	4	17	*p* = 0.42
Seldom	21	8	13	
Sometimes	12	3	9	
Often	3	0	3	
Always	2	0	2	
**Foot and ankle characteristics**				
Radiographic hallux valgus angle (°)	29.5 ± 8.0	31.1 ± 7.8	28.9 ± 8.0	2.2 (-2.5 to 6.8)
Manchester Scale (n)				
Grade 0 (none)	1	0	1	*p* = 0.57
Grade 1 (mild)	9	1	8	
Grade 2 (moderate)	26	7	19	
Grade 3 (severe)	24	8	16	
First MTP joint dorsiflexion (°)	83.8 ± 11.0	84.1 ± 9.7	83.6 ± 11.5	0.45 (-6.0 to 6.9)
Presence first MTP joint OA (n (%))	11 (18.3%)	3 (18.8%)	8 (18.2%)	*p* = 0.96
Presence forefoot callous (n (%))	44 (73.3%)	13 (81.3%)	31 (70.5%)	*p* = 0.40
Foot Posture Index (median (min, max))	7 (-3, 11)	7 (1, 11)	7 (-3, 11)	*p* = 0.93^c^
Dorsal arch height difference (mm)	12.5 ± 4.3	14.6 ± 5.0	11.8 ± 3.7	2.9 (0.47 to 5.3)*
Midfoot width difference (mm)	9.0 ± 3.5	9.2 ± 4.4	9.0 ± 3.2	0.27 (-1.8 to 2.3)
Foot Mobility Magnitude (mm)	15.8 ± 4.3	17.8 ± 5.2	15.1 ± 3.8	2.7 (0.22 to 5.2)*
Ankle lunge test (cm)	11.5 ± 3.1	11.5 ± 2.6	11.5 ± 3.3	-0.01 (-1.8 to 1.8)
Beighton scale (median (min, max))	1 (0, 9)	0 (0, 9)	1 (0, 6)	*p* = 0.42^c^
Paper grip test (n pass (%))	13 (21.7%)	1 (6.3%)	12 (27.3%)	*p* = 0.08
Hallux muscle strength (N)				
Plantarflexion	60.7 ± 28.3	51.6 ± 30.9	64.0 ± 26.8	-12.4 (-28.8 to 3.9)
Abduction	8.9 ± 6.6	7.5 ± 6.4	9.4 ± 6.7	-2.0 (-5.8 to 1.9)
Pressure-pain threshold (kPa)				
Medial first MTP joint	591.5 ± 231.4	552.5 ± 219.9	605.7 ± 236.3	-53.2 (-188.9 to 82.5)
Plantar first MTP joint	442.5 ± 193.1	428.9 ± 230.8	447.5 ± 180.3	-18.5 (-132.2 to 95.2)

MD: mean difference; CI: confidence interval; SF-36v2: Short Form 36 Health Survey, version 2.

*Significant difference between groups (*p* < 0.05) based on independent t-test (continuous variables) or Pearson’s Chi-squared test (dichotomous or categorical variables).

^a^Frequencies based on n = 59 due to missing data.

^b^Shoes worn to the examination session; due to footwear that could not be measured by our methods, relative heel height was based on n = 58, and relative ball width was based on n = 53.

^c^Based on Wilcoxon rank-sum test.

### Foot pain locations

Qualitative examination of foot pain locations revealed that the most common site of reported pain was the first MTP joint (n = 36, 60%), while pain in the lesser MTP joints (n = 16, 27%), hallux (n = 7, 12%) and lesser toes (n = 4, 7%) was also reported. Midfoot pain (n = 16, 27%) and heel pain (n = 7, 12%) were reported by some participants. Thirty-two individuals reported pain at one site, 18 reported pain at two sites, while six individuals reported pain at three different sites on the foot diagram.

### Associations with disabling foot pain

Sixteen participants were defined as having disabling foot pain [23]. Participant characteristics and foot and ankle characteristics for those with and without disabling foot pain are presented in Table 1. Those with disabling foot pain reported significantly poorer general health (mean difference (MD) -8.8, 95% CI: -17.3 to -0.29). Both DAH difference (MD 2.9 mm, CI: 0.47 to 5.3) and FMM (MD 2.7 mm, CI: 0.22 to 5.2) were significantly increased in those with disabling foot pain. Those with disabling foot pain were slightly older than those without disabling foot pain (MD 6.9 years, CI: -1.8 to 15.6) but this difference was not statistically significant. No other participant characteristics, foot and ankle characteristics or footwear factors showed significant differences between those with and without disabling foot pain. Notably, neither radiographic HV angle nor the presence of first MTP joint OA was found to be significantly associated with disabling foot pain.

### Univariate associations with foot pain VAS

Table 2 shows univariate associations between foot pain VAS, participant characteristics and foot and ankle characteristics. Lower educational attainment was associated with higher average foot pain VAS (p = 0.02), while poorer general health scores and higher work activity was associated with higher average and worst reported pain (p ≤ 0.05). Participants who wore shoes with a heel height > 25 mm to the examination session reported higher worst foot pain (p = 0.06), and those who failed the paper grip test reported higher average and worst foot pain (p < 0.05). An increased arch height difference between non weight-bearing and weight-bearing was associated with higher average and worst foot pain (p < 0.1), but interestingly lower Beighton scores indicating less generalised joint hypermobility were associated with higher average pain (p = 0.09). Correlations between foot pain and plantar pressure and force parameters are displayed in Table 3. The only in-shoe plantar pressure parameters that showed significant correlations with increasing foot pain were reduced force-time integrals under the first and second metatarsal heads (p < 0.1).

**Table 2:** Univariate associations between participant characteristics, foot and ankle characteristics and foot pain

	**Average pain VAS**		**Worst pain VAS**	
	**Univariate association**	***p *** **value**	**Univariate association**	***p *** **value**
**Participant characteristics**				
Gender	MD = 1.3 mm	0.85	MD = 6.9 mm	0.56
Age	Pearson’s r = 0.15	0.25	Pearson’s r = 0.06	0.66
BMI^a^	Pearson’s r = 0.07	0.61	Pearson’s r = 0.04	0.77
Ethnicity	ANOVA F = 0.12	0.88	ANOVA F = 0.16	0.85
Education	ANOVA F = 3.46	0.02*	ANOVA F = 1.50	0.22
General health score (SF-36v2)^a^	Pearson’s r = -0.32	0.01*	Pearson’s r = -0.26	0.05*
Physical activity level				
Work index	Pearson’s r = 0.32	0.02*	Pearson’s r = 0.34	0.009*
Sport index^a^	Pearson’s r = 0.05	0.73	Pearson’s r = 0.01	0.92
Leisure index	Pearson’s r = 0.00	0.98	Pearson’s r = -0.03	0.81
Footwear worn to examination				
Relative heel height > 25 mm	MD = 5.1 mm	0.46	MD = 20.6 mm	0.06*
Relative ball width	Pearson’s r = -0.01	0.97	Pearson’s r = -0.10	0.46
History of wearing high heels	MD = 6.3 mm	0.17	MD = 5.3 mm	0.49
Frequency of wearing high heels	ANOVA F = 1.07	0.38	ANOVA F = 0.40	0.81
**Foot and ankle characteristics**				
Radiographic hallux valgus angle	Pearson’s r = -0.02	0.88	Pearson’s r = 0.03	0.80
Manchester Scale (grade 0 to 3)	ANOVA F = 1.05	0.38	ANOVA F = 1.17	0.33
Presence first MTP joint OA	MD = 6.1 mm	0.31	MD = 5.9 mm	0.55
Presence forefoot callous	MD = 4.1 mm	0.44	MD = 3.7 mm	0.67
Foot Posture Index (score -12 to +12)	Spearman’s rho = 0.06	0.64	Spearman’s rho = -0.04	0.73
Dorsal arch height difference	Pearson’s r = 0.26	0.05*	Pearson’s r = 0.23	0.07*
Midfoot width difference	Pearson’s r = -0.12	0.41	Pearson’s r = -0.11	0.42
Foot Mobility Magnitude	Pearson’s r = 0.16	0.22	Pearson’s r = 0.14	0.29
First MTP joint dorsiflexion	Pearson’s r = -0.15	0.24	Pearson’s r = -0.01	0.93
Ankle lunge test	Pearson’s r = 0.07	0.59	Pearson’s r = 0.05	0.73
Beighton score (range 0 – 9)	Spearman’s rho = -0.22	0.09*	Spearman’s rho = -0.09	0.50
Paper grip test (pass/fail)	MD = 12.7 mm	0.02*	MD = 26.4 mm	0.003*
Hallux muscle strength				
Plantarflexion	Pearson’s r = -0.10	0.43	Pearson’s r = -0.10	0.44
Abduction^a^	Pearson’s r = -0.08	0.55	Pearson’s r = -0.01	0.92
Pressure-pain threshold				
Medial first MTP joint	Pearson’s r = -0.15	0.26	Pearson’s r = -0.14	0.30
Plantar first MTP joint	Pearson’s r = -0.12	0.38	Pearson’ r = -0.19	0.14

VAS: visual analogue scale; MD: mean difference; SF-36v2: Short Form 36 Health Survey, version 2.

*Statistically significant at alpha *p <* 0.1.

^a^Transformed (log, inverse or square).

**Table 3:** Correlations between worst and average foot pain VAS and plantar pressure and force parameters

	**Peak pressure (kPa)**					Pressure-time integral (kPa*s)				
	**M1**^**a**^	**M2**^**a**^	**M3-5**^**a**^	**Hallux**	**LT**	**M1**^**a**^	**M2**^**a**^	**M3-5**^**a**^	**Hallux**	**LT**^**a**^
Average pain	-0.08	0.02	0.07	0.14	0.07	-0.12	-0.07	0.01	0.03	-0.02
Worst pain	-0.01	-0.02	0.01	0.09	0.03	0.03	-0.01	0.05	0.06	-0.02
	**Maximum force (% BW)**					Force-time integral (% BW*s)				
	**M1**	**M2**	**M3-5**	**Hallux**	**LT**	**M1**	**M2**	**M3-5**	**Hallux**	**LT**
Average pain	-0.18	-0.20	-0.04	0.00	0.06	-0.23*	-0.23*	-0.14	-0.05	-0.00
Worst pain	0.08	-0.11	-0.01	-0.06	0.01	0.03	-0.10	-0.08	-0.08	-0.04

VAS: visual analogue scale; M: metatarsal head; LT: lesser toes;% BW: percentage body weight.

*Statistically significant at alpha *p <* 0.1.

^a^Log transformed.

### Multiple regression analysis

Multiple regression modelling (Table 4) showed that general health status, educational attainment, work activity index and force-time integral under the second metatarsal head explained 33% of the variance in average foot pain intensity over the past four weeks. As seen by the beta weights in Table 4, poorer general health, lower educational attainment, a higher work activity index, and lower second metatarsal force-time integrals were associated with increased average foot pain. With worst foot pain VAS as the dependent variable, the two significant contributors to the model were the paper grip test and work activity index, together explaining 20% of the variance in worst foot pain (Table 5). A failed paper grip test and higher work activity index were associated with increased worst foot pain. No other potential predictor variables were retained in the multiple regression models as they did not make a significant contribution (*p >* 0.05) (Tables 4 and 5). HV angle was a poor predictor of pain intensity when forced into the final model for average foot pain (0% change in R^2^, beta 0.007, *p* = 0.96) and worst foot pain (change in R^2^ 0.4%, beta 0.063, *p* = 0.61).

**Table 4:** **Multiple linear regression model**^**a **^**with average foot pain VAS as the outcome variable**

**Significant predictor variables**	**Standardised β weight**	***P *** **value**	**Multiple R**^**2**^
General health score (SF-36v2)	-0.296	0.01	0.330
Educational attainment	-0.232	0.05	
Work activity index	0.312	0.01	
Force-time integral (M2)	-0.269	0.02	
**Variables not retained in the model**			**Change in R**^**2**^
Paper grip test (pass = 0, fail = 1)	0.141	0.24	0.018
Dorsal arch height difference	0.108	0.38	0.010
Force-time integral (M1)	-0.106	0.47	0.007
Beighton Score	-0.144	0.22	0.020
**Variable forced into the model**			
Hallux valgus angle	0.007	0.96	0.000

VAS: visual analogue scale; SF-36v2: Short Form 36 Health Survey, version 2; M: metatarsal head.

^a^Cases excluded due to missing data (n = 3); therefore, analysis based on n = 57.

**Table 5:** **Multiple linear regression model**^**a **^**with worst foot pain VAS as the outcome variable**

**Significant predictor variables**	**Standardised β weight**	***P *** **value**	**Multiple R**^**2**^
Paper grip test (pass = 0, fail = 1)	0.312	0.02	0.204
Work activity index	0.250	0.05	
**Variables not retained in the model**			**Change in R**^**2**^
General health score (SF-36v2)	-0.188	0.12	0.035
Dorsal arch height difference	0.183	0.13	0.033
Relative heel height >25 mm	0.193	0.13	0.035
**Variable forced into the model**			
Hallux valgus angle	0.063	0.61	0.004

VAS: visual analogue scale; SF-36v2: Short Form 36 Health Survey, version 2.

^a^Cases excluded due to missing data (n = 2); therefore, analysis based on n = 58.

## Discussion

The intensity of foot pain experienced by individuals with HV is not determined by angular deformity or other passive structural factors, but rather is influenced by patient characteristics such as poorer general health status and increased occupational physical activity levels. In addition to patient factors, some dynamic foot and ankle characteristics were significantly associated with increasing foot pain in this study, such as hallux plantarflexion weakness determined by failure of the paper grip test, and reduced force-time integral under the second metatarsal during gait. In combination these factors explained approximately 20 to 30% of the variance in foot pain associated with HV. Clearly, a large proportion (approximately 70 to 80%) of the variance remains unexplained by the comprehensive set of factors investigated in this study, and further research is warranted to explore the complexity of foot pain associated with HV.

Our investigation included several structural foot and ankle characteristics frequently assessed by clinicians, including HV angle, radiographic signs of first MTP joint OA, forefoot callous, foot posture and passive joint ROM. Interestingly, participants with first MTP joint OA (n = 11) were not more likely to report disabling foot pain (Table 1) or greater foot pain intensity (Table 2). This finding is consistent with previous studies [13,28], although foot pain is often attributed to degenerative arthritic processes [17]. While forefoot callous is another factor often considered to be correlated with foot pain, both our study and a study by Spink et al. [16] show no association between the presence of plantar hyperkeratotic lesions and foot pain. Furthermore, footwear factors were not significantly associated with disabling foot pain in our study. Having a relative heel height of greater than 25 mm was potentially associated with worst pain VAS in our univariate analysis (*p =* 0.06), but no footwear variables made a significant contribution to the multivariate regression models.

Dynamic foot and ankle assessments may be more significant than static structural measures in explaining foot pain associated with HV. Failure of the paper grip test helped explain worst foot pain in our multivariate modelling (Table 5). It is plausible that motor weakness surrounding the hallux may occur in response to pain [44]. For example, a previous study by Mickle et al. (n = 312) [3] reported reduced hallux flexor strength in older adults with disabling foot pain compared to those without disabling foot pain. Further investigation is warranted, as our study sample size may not have provided sufficient power to detect a significant difference in muscle strength between those with and without disabling foot pain (Table 1). In-shoe plantar pressure analysis showed an inverse correlation between average foot pain and force-time integrals under the first and second metatarsal heads (Table 3). Given that the most common site of reported pain was the first MTP joint (60%), it is plausible that those with more painful feet might walk more cautiously and spend less time loading the medial forefoot when wearing shoes, although other studies have linked higher barefoot plantar pressures to pain in HV [15]. The lack of standardised footwear in our study is an important consideration, as the observed pressures could also reflect characteristics of the footwear. Further studies investigating barefoot versus in-shoe plantar pressures profiles in HV are warranted.

Our study found that lower educational attainment was associated with higher average pain levels using univariate and multivariate analysis. Cho et al. [7] (n = 563) previously reported lower educational attainment to be associated with painful HV using univariate analysis methods. It is plausible that lower educational attainment could be associated with more physically demanding work, which would be consistent with our finding of a correlation between foot pain and occupational physical activity levels (Table 2). Other studies have reported foot pain to be associated with increasing age [18], female sex [3,7,18] and higher BMI [3,13,19,20], although none of these associations were apparent in our sample of adults with HV.

It is evident that individuals with HV experience varying levels of foot pain, thus different pain scales and definitions used in previous studies could help explain inconsistent reports. Sixteen out of 60 participants in our study reported disabling foot pain [23], while four individuals reported no foot pain at all, leaving 40 individuals with some degree of mild to moderate foot pain. We chose to use a relatively strict definition (at least one item on the Manchester Foot Pain and Disability Index reported on “most/every day”) [23] to differentiate those individuals with disabling foot pain (n = 16) from those without (n = 44). In addition we used a simple pain VAS to establish pain intensity on a continuous scale. The complex and multidimensional nature of pain, which may be only partially captured by a single-item measure such as the pain VAS, should be acknowledged [45]. Another point to note is that study participants were asked about foot pain, not “big toe pain”, which may be more specific to HV. Although the first MTP joint was the most common site of reported pain, some participants reported pain at another site such as the arch or heel, which may not have been directly related to HV. Nonetheless, this is the first study to examine both foot pain intensity and disabling foot pain in HV. Future studies should utilise detailed measures of pain that capture the complexity of this construct in populations with HV.

Several considerations are relevant when interpreting our study findings. The sample size in our investigation was somewhat limited due to the scope of radiographic and clinical measurements obtained, and this may impact on the generalisability of our findings. Our sample size was determined by a priori power calculations and was considered adequate to investigate up to six predictor variables in a multiple regression model. While previous studies have found that painful HV is associated with female sex [7], the number of males in our study was small (n = 7), which may explain why our study did not find an association between foot pain and female sex. While HV is approximately 2.3 times more prevalent in females compared to males in the general population [1], the gender bias in our study was more pronounced, which may indicate that women were more likely to volunteer to participate. Finally, due to the cross-sectional design of this study, conclusions cannot be drawn regarding which factors may contribute to the development of foot pain, and which factors may develop as a consequence of pain.

## Conclusions

Severity of hallux deviation and radiographic signs of first MTP joint OA are poor predictors of foot pain in HV. Foot pain and disability associated with HV must be evaluated in the context of patient characteristics such as general health status and occupational physical activity levels. Our data suggest that assessment of static foot posture and joint ROMs may be less helpful in guiding management decisions for painful HV, therefore clinical examination of HV should include assessment of dynamic factors such as hallux plantarflexion strength and gait parameters. Due to the high proportion of people with HV who experience foot pain (93% of our sample), this is an important area for research, yet a large proportion of the variability of pain in HV remains unexplained. Further research is warranted to understand functional adaptations to foot pain, and to investigate potentially modifiable factors that may contribute to the development of foot pain in HV. Conservative management could then be appropriately targeted to address factors contributing to foot pain and disability in HV.

## Competing interests

The authors declare that they have no competing interests.

## Authors’ contributions

SH carried out participant recruitment and data collection, conducted statistical analysis and drafted the manuscript. BV and MS were involved with study conception and design, interpretation of data and critical revision of the manuscript. All authors read and approved the final manuscript.
